# Preliminary Investigation of the Antioxidant, Anti-Diabetic, and Anti-Inflammatory Activity of *Enteromorpha intestinalis* Extracts

**DOI:** 10.3390/molecules26041171

**Published:** 2021-02-22

**Authors:** Biswajita Pradhan, Srimanta Patra, Chhandashree Behera, Rabindra Nayak, Bimal Prasad Jit, Andrea Ragusa, Mrutyunjay Jena

**Affiliations:** 1 Algal Biotechnology and Molecular Systematic Laboratory, Post Graduate Department of Botany, Berhampur University, Bhanja Bihar, Berhampur 760007, India; pradhan.biswajita2014@gmail.com (B.P.); chhandashreebehera@gmail.com (C.B.); rabindran335@gmail.com (R.N.); 2 Cancer and Cell Death Laboratory, Department of Life Science, National Institute of Technology Rourkela, Rourkela 769008, India; 518LS2007@nitrkl.ac.in; 3 Department of Biochemistry, All India Institute of Medical Science, Ansari Nagar, New Delhi 110023, India; bimaljit2019@gmail.com; 4 Department of Biological and Environmental Sciences and Technologies, Campus Ecotekne, University of Salento, via Monteroni, 73100 Lecce, Italy; 5 CNR-Nanotec, Institute of Nanotechnology, via Monteroni, 73100 Lecce, Italy

**Keywords:** oxidative stress, reactive oxygen species, antioxidant, polyphenols, diabetes, anti-inflammatory, marine algae, seaweed

## Abstract

Marine algae are a promising source of potent bioactive agents against oxidative stress, diabetes, and inflammation. However, the possible therapeutic effects of many algal metabolites have not been exploited yet. In this regard, we explored the therapeutic potential of *Enteromorpha intestinalis* extracts obtained from methanol, ethanol, and hexane, in contrasting oxidative stress. The total phenolic (TPC) and flavonoids (TFC) content were quantified in all extracts, with ethanol yielding the best values (about 60 and 625 mg of gallic acid and rutin equivalents per gram of extract, respectively). Their antioxidant potential was also assessed through DPPH^•^, hydroxyl radical, hydrogen peroxide, and superoxide anion scavenging assays, showing a concentration-dependent activity which was greater in the extracts from protic and more polar solvents. The α-amylase and α-glucosidase activities were estimated for checking the antidiabetic capacity, with IC_50_ values of about 3.8 µg/mL for the methanolic extract, almost as low as those obtained with acarbose (about 2.8 and 3.3 µg/mL, respectively). The same extract also showed remarkable anti-inflammatory effect, as determined by hemolysis, protein denaturation, proteinase and lipoxygenase activity assays, with respectable IC_50_ values (about 11, 4, 6, and 5 µg/mL, respectively), also in comparison to commercially used drugs, such as acetylsalicylic acid.

## 1. Introduction

Secondary metabolites, such as polyphenols, carotenoids, terpenes, and alkaloids are gaining an ever-increasing attention because of their antioxidant capacity and the consequent beneficial effect on the human body [1,2]. These bioactive compounds have been widely investigated in plants and fruits, but they still represent a largely unexplored source of phenolic and flavonoid compounds in marine algae. Their extraction is usually accomplished in organic solvents, such as petroleum ether, hexane, chloroform, ethyl acetate, acetone, and methanol. Nevertheless, cheap and non-toxic solvents are preferred from an industrial point-of-view. In addition, alcoholic extracts generally allow a better extraction of polyphenols [3]. Because of their well-known beneficial health effects, as well as their positive influence on several pathologies, such as cancer, diabetes, cardiovascular, neurodegenerative, and inflammatory diseases, and in order to improve their pharmacokinetics, secondary metabolites are also being exploited in more technological formulations [4,5].

Marine algae have been extensively used as food for humans and animals. Macroalgae are rich in minerals, vitamins, polysaccharides, and other bioactive substances, such as proteins, lipids, carotenoid, and polyphenols. These latter compounds are produced by marine algae and sea grasses primarily for protective, structural, and ecological objectives; nevertheless, they also confer interesting properties to their raw material, such as antioxidant, antidiabetic, anti-inflammatory, antiviral, antibacterial, and other disease-preventive agents [6]. The beneficial health effects of marine macroalgae are largely related to their radical-scavenging ability, which can reduce oxidative stress. The commercialization of seaweed is based on their use as food supplements and nutraceuticals, ultimately developing non-toxic functional products with antioxidant properties.

Green seaweeds have been shown to possess strong antioxidant properties due, among others, to the contained flavonoids, bromophenols, and chlorophylls [7,8]. Among them, the *Enteromorpha* genus belongs to the class Chlorophyceae and it represents a rich source of secondary metabolites. For example, alcoholic extracts from *E. compressa* were recently shown to possess strong antioxidant capacity, also because of the high content of phenols and flavonoids (about 98 and 562 mg of gallic acid and rutin equivalents per gram of dry weight of extract, respectively) [9]. Interestingly, Wekre and colleagues studied the polyphenolic content in *E. intestinalis* by different techniques, e.g., NMR and RP-HPLC, also determining the major constituents by mass analysis [10]. Nevertheless, this group of algae also possess a variety of additional health-beneficial effects, e.g., anti-tumoral, antibacterial, and antidiabetic, that deserve further investigation [11,12,13,14,15].

Diabetes mellitus is a chronic metabolic disorder characterized by high blood glucose levels and it can lead to renal dysfunction, cardiovascular diseases, and retinal damage [16]. Commercially available antidiabetic drugs exert several adverse side effects, pushing the research for novel natural drugs. Marine algal bioactive compounds were able to inhibit α-amylase, α-glucosidase, dipeptidyl peptidase-4, aldose reductase, and protein tyrosine phosphatase 1B (PTP 1B) enzymes, which play a key role in the digestion of carbohydrate, thus leading to delayed glucose absorption in blood and plasma [17]. Marine algal compounds were also able to modulate GLUT-4 and AMPK signaling pathways and trigger glucose tolerance. The α-amylase inhibition delays the assimilation process by hindering the breakdown of starch and can be used as a target for treatment against hyperglycemic disorder [17].

Inflammation is usually referred to as an intricate series of biological reactions of the vascular tissues subjected to deleterious stimuli. In addition, inflammation is also associated with pain that leads to protein denaturation, increase of membrane modifications, and vascular permeability [18]. Non-steroidal anti-inflammatory drugs (NSAIDs) have been extensively used as anti-inflammatory agents although they present several adverse effects, such as gastric irritation, which can lead to gastric ulcer [19]. The marine environment is rich of both micro- and macro-algal biodiversity, which could contain novel therapeutic compounds [20]. Hence, the identification and screening of natural bioactive compounds from marine algal source with anti-inflammatory activity is promising.

Although some studies already described the antioxidant capacity of marine algae, the ability of bioactive compounds in *Enteromorpha intestinalis* in fighting free radicals and related diseases, e.g., diabetes and inflammatory diseases, are yet to be investigated.

The aim of this study was to investigate the ability of methanol, ethanol, and hexane extracts of *Enteromorpha intestinalis* in scavenging free radicals through several in vitro assays, such as DPPH^•^, hydroxyl radical, H_2_O_2_ radical, and superoxide anion radical scavenging assay. In addition, a preliminary investigation of their anti-diabetic activity was performed via α-amylase and α-glucosidase inhibition assays. On the other hand, inhibition of albumin denaturation, antiproteinase activity, membrane stabilization, and anti-lipoxygenase activity were used to evaluate the potential anti-inflammatory capacity of the extracts as compared to commercial acetylsalicylic acid.

## 2. Results

### 2.1. Solvent Extracts of E. intestinalis Are Promising Source of Antioxidative Phytochemicals

Three different solvents were tested for the extraction of the secondary metabolites from *E. intestinalis*, namely methanol, ethanol, and hexane, yielding the corresponding MEEI, EEEI, and HEEI extracts. A qualitative phytochemical analysis revealed the presence of glycosides, alkaloids, coumarin, terpenoids, proteins, phenols, and tannins (Table 1).

**Figure 1:**
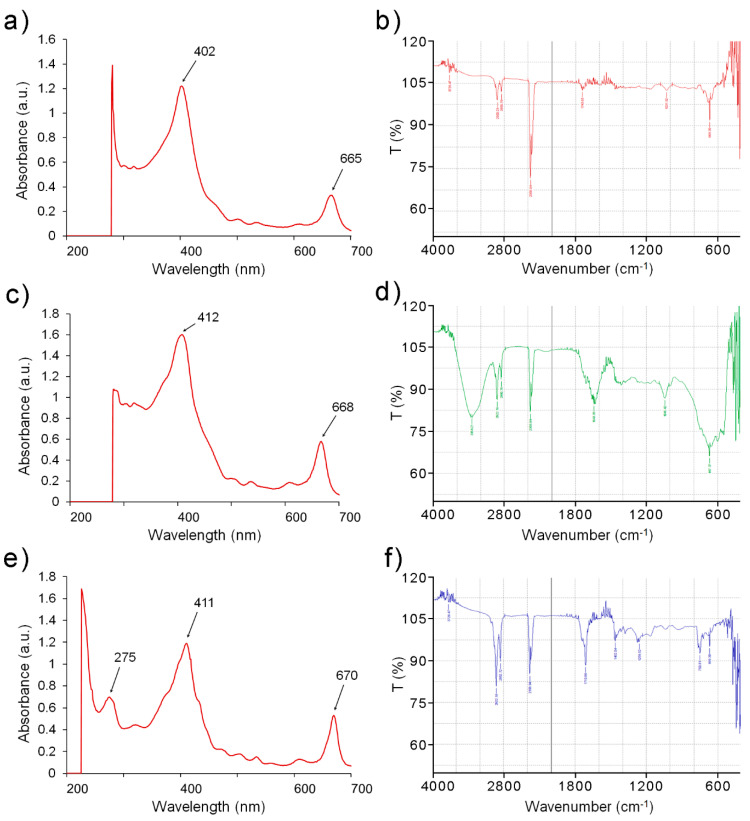
UV-Vis (**a**,**c**,**e**) and FT-IR (**b**,**d**,**f**) spectral analyses of the extracts of *Enteromorpha intestinalis* obtained by methanol (**a**,**b**), ethanol (**c**,**d**), and hexane (**e**,**f**). The analyses evidenced minor differences among the spectra corresponding to the different concentration and composition of the bioactive functional groups extracted.

No steroids, anthocyanins, and reducing sugars were recovered by any solvent, while saponins were detected only in the methanolic extract. The qualitative test was followed by a UV-visible spectrophotometric analysis that evidenced absorption peaks between 260–290, 380–460, and 650–680 nm (Figure 1a,c,e).

The presence of functional groups typical from flavonoids and other phenolic compounds was also established by FT-IR analysis (Figure 1b,d,f), and the major peaks observed in each extract are summarized in Table 2.

To obtain a quantitative indication of the presence of these secondary metabolites, the TPC and TFC contents in MEEI, EEEI, and HEEI were determined. The TPC was found to be higher in ethanolic extract (about 60 mg of GAE/g) while the lowest values were obtained from the hexane extract. Similarly, the TFC ranged from about 625 mg of RUE/g in EEEI to about 152 mg of RUE/g in the hexane extract. Much lower amounts of ascorbic acid were detected in all extracts, although with a similar trend. The total antioxidant capacity (TAC) exerted by the ethanolic extracts was quantified to be about 177 mg of AAE/g, as determined by the phosphomolybdenum assay. Again, the lowest TAC was observed in the hexane extract (about 45 mg of AAE/g) (Table 3).

**Figure 2:**
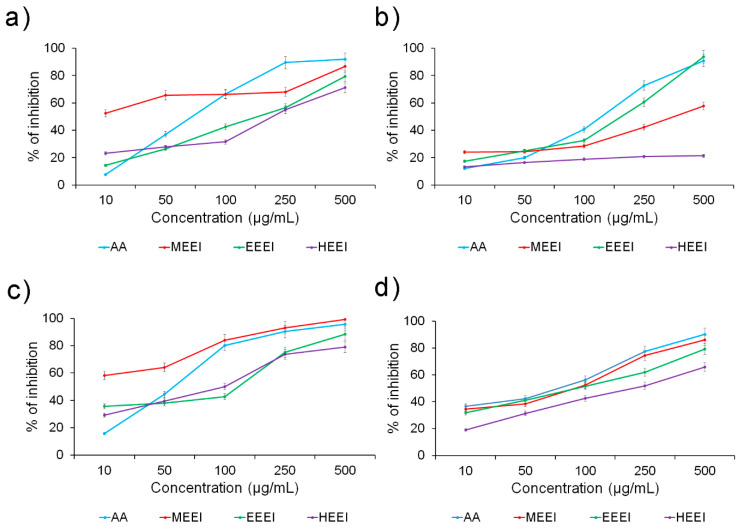
Antioxidant scavenging capacity of the different solvent extracts from *Enteromorpha intestinalis* as determined by (**a**) DPPH^•^ radical scavenging activity, (**b**) hydrogen peroxide (H_2_O_2_) scavenging activity, (**c**) hydroxyl (^•^OH) ion radical scavenging activity, and (**d**) superoxide (^•^O_2_) scavenging activity assays.

Phenols and flavonoids exhibit potent antioxidant and free radical scavenging activity. The ROS-associated free radical scavenging properties of MEEI, EEEI, and HEEI were measured by DPPH^•^, hydroxyl radicals, hydrogen peroxide, and superoxide radicals scavenging assay in the range 10–500 µg/mL and compared to ascorbic acid (AA), used as reference (Figure 2).

In general, an increasing antioxidant effect was noted by increasing the extracts’ concentration in all assays. In the DPPH^•^ radical scavenging assay, the MEEI extract exhibited the highest scavenging activity at lower concentrations (up to 50 µg/mL) in comparison to the other extracts and ascorbic acid, although this difference diminished at higher concentrations (Figure 2a). MEEI and even more EEEI also had better ability to scavenge H_2_O_2_ radicals compared to HEEI, especially at concentrations above 100 µg/mL (Figure 2b). The MEEI extract presented the best inhibition profile in the hydroxyl ion radical assay, exhibiting even better values than ascorbic acid, especially at concentrations below 100 µg/mL (Figure 2c). On the other hand, much closer data were obtained when evaluating the superoxide radical scavenging potency, although the HEEI extract showed again the worst inhibition values (Figure 2d).

Similar results were obtained by comparing the IC_50_ values, where the MEEI extract showed very low inhibitory concentrations in the DPPH^•^ and hydroxyl radical scavenging assays (about 0.5 and 0.3 µg/mL, respectively), also in comparison to ascorbic acid (about 2.6 and 2.3 µg/mL, respectively) (Table 4). Satisfactory values were also obtained by the EEEI extract, with concentrations comparable to that of ascorbic acid in the H_2_O_2_ scavenging assay (IC_50_ values of about 3.2 and 3.1 µg/mL, respectively). On the other hand, the extract from the apolar hexane yielded the worst IC_50_ values in all assays.

The correlation between TPC and antioxidant activity was calculated through linear regression for all the extracts. As expected, high correlation coefficients were obtained in all cases (R^2^_(DPPH^•^)_ = 0.93 and R^2^_(H2O2)_ = 0.99 for the ethanolic extract; R^2^_(DPPH^•^)_ = 0.87 and R^2^_(H2O2)_ = 0.99 for the methanolic extract; R^2^_(DPPH^•^)_ = 0.97 and R^2^_(H2O2)_ = 0.68 for the hexane extract) confirming the important role of polyphenols in contrasting the oxidative stress.

### 2.2. Solvent Extracts of E. intestinalis Exhibit Potential Antidiabetic Activity

**Figure 3:**
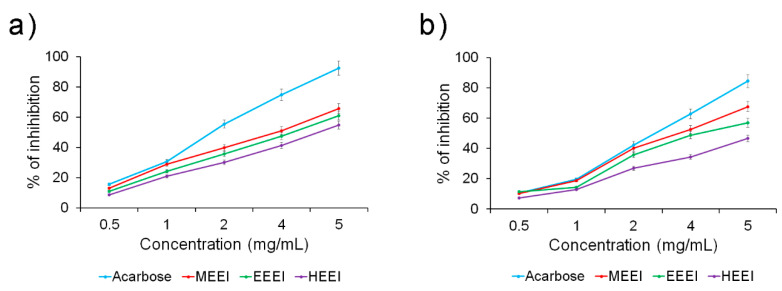
Antidiabetic capacity of different solvent extracts from *Enteromorpha intestinalis* as determined by (**a**) α-amylase, and (**b**) α-glucosidase inhibitory activity assays.

To determine the antidiabetic property of the solvent extracts of *E. intestinalis*, their ability to inhibit α-amylase and α-glucosidase activities were investigated. MEEI, EEEI, and HEEI were shown to have a concentration-dependent inhibitory effect on the α-amylase activity, with the MEEI extract displaying the best inhibitory effect (Figure 3a).

The α-glucosidase activity also followed a similar pattern, although the difference between the MEEI extract and the other ones was even more pronounced (Figure 3b). The potent α-amylase and α-glucosidase activity of the MEEI extract was confirmed by very good IC_50_ values (about 3.8 µg/mL in both assays), quite close to those obtained with acarbose, used as reference (IC_50_ of about 2.8 and 3.3 µg/mL, respectively) (Table 5). Slightly higher values were obtained by EEEI and, especially, HEEI extracts.

### 2.3. Solvent Extracts of E. intestinalis Are Promising Sources of Anti-Inflammatory Compounds

**Figure 4:**
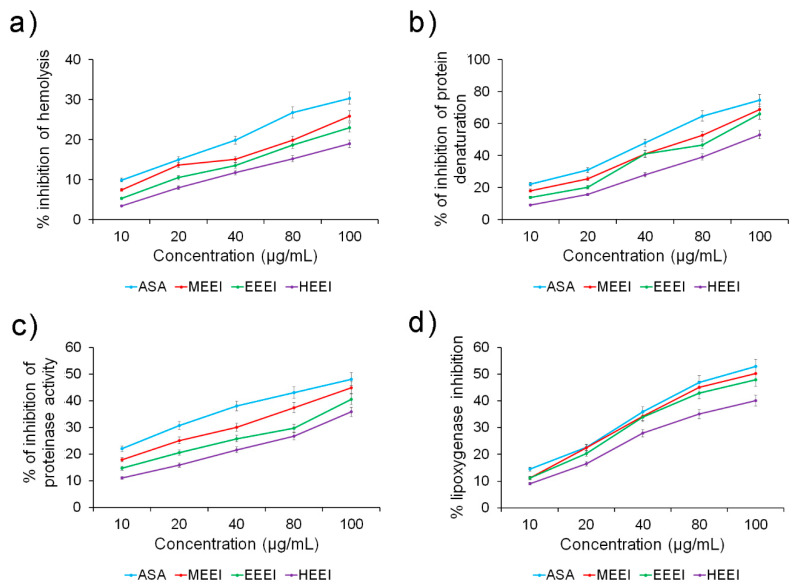
Anti-inflammatory capacity of different solvent extracts from *E. intestinalis* as determined by (**a**) hemolysis assay, (**b**) protein denaturation, (**c**) proteinase inhibitory activity, and (**d**) lipoxygenase inhibitory activity.

Four assays were performed to determine the anti-inflammatory capacity of the *E. intestinalis* extracts, namely hemolysis, protein denaturation, proteinase activity, and lipoxygenase activity. All extracts were able to inhibit all the activities in a concentration-dependent manner (Figure 4). Again, the MEEI extract was the best inhibition, followed by EEEI. The difference between the two polar extracts was almost non-existent in inhibiting lipoxygenase, where they also displayed an activity comparable to that of acetylsalicylic acid (ASA), used as reference (Figure 4d). On the other hand, the HEEI extract resulted to be the least powerful inhibitor at all the tested concentrations.

The corresponding IC_50_ values were estimated by linear regression in all assays, confirming the very good performance of the MEEI extract, followed closely by EEEI and, to a greater extent, by HEEI (Table 6). The IC_50_ values of the alcoholic extracts were almost comparable to those obtained by ASA, especially in inhibiting protein denaturation, proteinase activity, and lipoxygenase activity (IC_50_ values of about 3.7, 5.8, and 4.7 µg/mL for MEEI compared to 3.1, 5.1, and 4.5 for ASA, respectively). On the other hand, greater differences were noted in inhibiting hemolysis compared to ASA (IC_50_ value of about 8.6 µg/mL), although the extracts still exhibited satisfactory values, especially MEEI (IC_50_ value of about 10.8 µg/mL).

## 3. Discussion

Recently, phytochemicals have attained great attention for the screening and development of novel therapeutic pharmacophores [21]. Algae-based bioactive compounds, such as alkaloids, carbohydrates, proteins, glycosides, flavonoids, phenols, saponins, tannins, coumarins, and terpenoids, have shown promising in vitro results and could be extensively used as therapeutic agents against several diseases [17]. The present investigation was designed to understand the in vitro antioxidant potency of different solvent extracts of *Enteromorpha intestinalis*, a marine alga typical from Chilika lagoon, in India, with particular attention to their antidiabetic and anti-inflammatory activity. Three solvents with different polarity and physico-chemical characteristics, namely methanol, ethanol, and hexane, were used to extract many secondary metabolites from the plant. More in detail, alkaloids, glycosides, steroids, coumarins, terpenoids, proteins, coumarins, phenols, and tannins were detected in all extracts, while saponins were present only in the methanolic one, thus contributing to their antioxidant and disease-preventing potency [9].

Flavonoids, and polyphenols in general, are abundantly present in many plants, fruits, and derived products, and they exert an antioxidant effect by neutralizing free radicals [22,23,24,25]. The UV-visible and the FT-IR spectrophotometric analyses also yielded the typical signals of phenolic compounds, thus confirming their presence also in the *E. intestinalis* extracts [26]. The results of our study are in the line with the findings from a previous study in *Padina pavonica* [27] and *Sargassum wightii* seaweed extracts [28]. A quantitative analysis revealed that the highest content of phenols and flavonoids was present in the EEEI (about 60 and 625 mg of GAE and RUE per gram of extract for TPC and TFC, respectively), closely followed by the MEEI (about 23 and 416 mg of GAE and RUE per gram of extract for TPC and TFC, respectively). On the other hand, the hexane extract contained smaller amounts (about 12 and 152 mg of GAE and RUE per gram of extract for TPC and TFC, respectively), probably because the apolar solvent was not as efficient as the protic solvents in extracting these molecules. These data are in line with previous findings on the *E. compressa*, were the alcoholic solvents also extracted slightly higher but still comparable amounts of TPC and TFC (about 98 and 562 mg of GAE and RUE per gram of extract, respectively) [9]. The higher content of polyphenols in the ethanolic extract also resulted in the best TAC (about 177 mg of AAE per gram of extract), somewhat lower compared to the corresponding methanolic extract from *E. compressa* (about 339 mg of AAE per gram of extract) [9].

The aberrant accumulation of H_2_O_2_ is liable for oxidative stress [29,30] and inflammatory reactions, which can contribute to the development of pathological conditions, such as cancer, diabetes, and cardiovascular diseases [31]. The rapid decomposition of H_2_O_2_ subsequently generate hydroxyl radicals (^•^OH) that initiate lipid peroxidation and cellular damage [32]. The present study revealed potent inhibition of H_2_O_2_ radicals by the extracts in a concentration-dependent manner, with lower IC_50_ values obtained from the alcoholic extracts (about 4.7 and 3.2 µg/mL for the MEEI and EEEI extracts, respectively) compared to that from the apolar one (about 18.4 µg/mL). This data is in agreement with that achieved from the methanolic extract from *E. compressa* (about 2.8 µg/mL) [9] but considerably lower than that from the root of *Asparagus racemosus* Linn (about 27.6 µg/mL) [33]. Superoxide anion radicals are well known initiators of hydrogen peroxide or singlet oxygen in living systems [34]. Again, all the extracts from *E. intestinalis* displayed good superoxide radical scavenging activity in a concentration-dependent manner but with lower IC_50_ values for MEEI and EEEI (about 2.5 and 2.7 µg/mL, respectively) compared to HEEI (3.69 µg/mL) and confirming our previous findings [9,35]. A similar trend was also obtained by the DPPH^•^ and in the hydroxyl radical scavenging assays. DPPH^•^ is a stable organic free radical, which loses its absorption by accepting an electron or a free radical species. By getting an electron from an antioxidant molecule, DPPH^•^ becomes diamagnetic by reduction into hydrazine derivative [36]. The MEEI extract presented very low IC_50_ values (about 0.5 and 0.3 µg/mL for the DPPH^•^ and the ^•^OH scavenging assays, respectively) performing significantly better than ascorbic acid (about 2.6 and 2.3 µg/mL for the DPPH^•^ and the ^•^OH scavenging assays, respectively) and compared to the extracts from *E. compressa* (about 3.2 and 2.9 µg/mL, respectively) and *Asparagus racemosus* Linn (about 9.9 and 169.3 µg/mL, respectively) [33].

Diabetes mellitus (DM) is a metabolic disorder that affects the global population in the modern era [37,38]. The acute phase of diabetes is correlated with oxidative stress, which triggers nephropathy, retinopathy, and neuropathy [16]. However, hyperglycemia can be suppressed via α-amylase inhibition, which allows D-glucose absorption into the bloodstream [39]. Acarbose has been found to be an efficient drug against type 2 diabetes by suppressing the hydrolysis of carbohydrates [40]. MEEI, EEEI, and HEEI exhibited potential anti-diabetic effects, as suggested by their ability to inhibit α-amylase activity with low IC_50_ values (about 3.8, 4.2, and 4.7 µg/mL, respectively), although slightly higher than that from acarbose at the same concentration (about 2.8 µg/mL) [41]. Nevertheless, this difference was less marked when testing the α-glucosidase inhibitory activity, especially for the MEEI extract (IC_50_ of about 3.8 and 3.3 µg/mL for MEEI and acarbose, respectively).

Leukocytes play a pivotal role in cellular infiltration during inflammation process by releasing lysosomal enzymes, such as proteases, which cause tissue damage [42]. Furthermore, inflammation triggers secondary damages via free radical-induced lipid peroxidation. As the lysosomal membrane is similar to red blood cell membrane, hemolysis inhibition provides a good insight into the inflammation process. In fact, the stabilization of the cell membrane inhibits the lysis and release of cytoplasmic material, thus stopping the inflammation [43]. The MEEI was the most efficient in protecting against heat-induced hemolysis, although less successfully than ASA (IC_50_ of about 10.8 and 8.6 µg/mL, respectively) but better than an aqueous extract from *Albuca setosa* (14% inhibition at 125 µg/mL) [42]. All *E. intestinalis* extracts, especially the MEEI, also inhibited protein denaturation, a typical process characterizing inflammation, with low IC_50_ values (about 3.7 and 3.1 µg/mL for MEEI and ASA, respectively), much more efficiently than a water extract from the stem of *Wedelia trilobataon* (about 51.1 µg/mL) [44]. Similar results were obtained in inhibiting proteinase and lipoxygenase activity. Lipoxygenases are key enzymes which catalyze deoxygenation of polyunsaturated fatty acids to produce essential mediators of *cis*- and *trans*-conjugated diene hydroperoxides, such as the leukotrienes. The EEEI and especially the MEEI extracts possessed high concentration-dependent proteinase and lipoxygenase inhibitory activities, as already observed in previous studies with *Leptadenia pyrotechnica* and *Mahonia aquifolium* extracts [45,46], with IC_50_ values (about 4.7, 5.0, and 6.0 µg/mL for MEEI, EEEI, and HEEI, respectively) almost comparable to that of acetylsalicylic acid (about 4.5 µg/mL), thus confirming their potential anti-inflammatory effect.

## 4. Materials and Methods

### 4.1. Sampling

A sample of the marine macroalgae *Enteromorpha intestinalis* was collected in June 2019 by hand picking from the Chilika lagoon (Kalijai), in India (longitude 19°39.934′ N, latitude 85°13.033′ E). After morphological characterization, the characteristics of the sample were compared with those in the monograph of marine algae and the alga identified as *Enteromorpha intestinalis* [47]. The sample was 25 cm in height and its cells were 10–14 µm long and 6–10 µm wide. The specimen was deposited in the Herbarium of the Department of Botany, Berhampur University, Odisha (India), and assigned the herbarium number BU1919.

### 4.2. Extract Preparation

The sample was dried at 80 °C in an oven, crushed to powder with a mechanical grinder, and extracted through a Soxhlet apparatus. After the extraction in different solvents, the filtrate sample was subjected to evaporation in a rotary evaporator and the % of yield of *E. intestinalis* calculated. The three solvent extracts, i.e., the methanolic extract of *E. intestinalis* (MEEI), the ethanolic extract of *E. intestinalis* (EEEI), and the hexane extract of *E. intestinalis* (HEEI), were prepared as already described [35]. The dried extracts were kept at 4 °C before use.

### 4.3. UV Visible and FT-IR Spectroscopy

The UV-visible spectra of the different solvent extracts from *E. intestinalis* were recorded from 200 to 700 nm on an Eppendorf BioSpectrometer 6136 spectrophotometer. FT-IR analyses were recorded using a Nicolet FT-IR spectrophotometer in transmittance mode from 400 to 4000 cm^−1^.

### 4.4. Qualitative Phytochemical Screening

Qualitative analysis of MEEI, EEEI, and HEEI extracts was performed to conclude the presence of various bioactive metabolites using the standard qualitative procedures as already reported elsewhere [35].

### 4.5. Estimation of Total Phenol Content and Total Flavonoids

The total phenolic content of the MEEI, EEEI, and HEEI was estimated by the Folin–Ciocalteu method by measuring the optical density (OD) at 765 nm, as already reported [48]. Gallic acid was used as internal standard. The results were expressed as milligram of gallic acid equivalent dry weight of the sample per 1 g of extract (mg of GAE/g).

### 4.6. Estimation of Total Flavonoid Content

The spectrophotometric determination of total flavonoid content was estimated by measuring the OD at 415 nm, as already reported [49]. Rutin was used as internal standard. The TFC of the extract was expressed in terms of rutin equivalent dry weight of the algal sample per 1 g of extract (mg of RUE/g).

### 4.7. Estimation of Ascorbic Acid Content

The ascorbic acid content was estimated following a procedure by Al-Ani et al. [50]. Briefly, the crude extract (1 mL) was mixed with 2,4-nitro-phenylhydrazine reagent (1 mL) and incubated at 95 °C for 15 min. Then, H_2_SO_4_ (85%, 5 mL) was added dropwise to the reaction mixture in ice cold condition. After 30 min of incubation, the absorbance was measured at 520 nm with an Eppendorf BioSpectrometer 6136 (Eppendorf AG, Hamburg, Germany). Ascorbic acid was used as standard and its content in the extracts was expressed in terms of µg of AA per 1 g of extract (µg of AA/g).

### 4.8. Determination of Free Radical Scavenging Activity

#### 4.8.1. 1,1-Diphenyl-2-picrylhydrazyl Radical (DPPH^•^) Scavenging Activity

The DPPH^•^ free radical scavenging activity of the MEEI, EEEI, and HEEI extracts was determined as already described (OD at 517 nm) by using ascorbic acid as standard [35]. The DPPH^•^ decolorization was measured and expressed as % of inhibition.

#### 4.8.2. Hydroxyl Radical Scavenging Activity

The hydroxyl radical scavenging efficacies of different solvent extracts were determined as already described (OD at 562 nm) by using ascorbic acid as standard [35]. The scavenging potency was calculated and represented as % of inhibition.

#### 4.8.3. Superoxide Anion Radical Scavenging Activity

The superoxide anion radical scavenging efficacy of the different solvent extracts was determined as already described (OD at 560 nm) by using ascorbic acid as standard [9]. The scavenging potency was measured and represented as % of inhibition.

#### 4.8.4. Hydrogen Peroxide Radical Scavenging Activity

The hydrogen peroxide radical scavenging efficacy of the different solvent extracts was determined as already reported (OD at 230 nm) by using ascorbic acid as the standard [51]. The scavenging potency was measured and represented as % of inhibition.

#### 4.8.5. Total Antioxidant Activity by Phosphomolybdenum Assay

The total antioxidant activity of different solvent extracts was determined by measuring the OD at 695 nm, as already reported [22]. Ascorbic acid was used for preparing the calibration curve. The total antioxidant activity of the extracts was expressed as the number of gram equivalents of ascorbic acid.

### 4.9. Antidiabetic Activity

#### 4.9.1. Determination of α-Amylase Inhibition

The α-amylase inhibitory activity was measured according to previously published procedures [39,52]. Briefly, stock sample solutions were prepared (10 mg/mL) and further diluted with 20 mM phosphate buffer saline (PBS) at pH 6.9 to a final range of 0.5–5.0 mg/mL. The sample (20 μL) was mixed with 20 mM PBS (20 μL, pH 6.9) and 1% starch solution (20 μL). The reaction mixture was incubated in an orbital shaker (37 °C, 3 min) followed by the addition of porcine pancreatic α-amylase solution (20 μL). The reaction was incubated again (37 °C, 15 min). The reaction was then stopped by adding 1 M HCl (20 μL) and iodine test solution (100 μL, 2.5 mM) and the absorbance of the solution was measured at 630 nm. The α-amylase inhibition was calculated and expressed as %. The 50% inhibitory concentration (IC_50_) of α-amylase was calculated through linear regression and the percentage of inhibition calculated according to the following equation:

% inhibition = [1 − (Ab_2_ − Ab_1_)/(Ab_4_ − Ab_3_)] × 100, (1)

where Ab_1_ = absorbance of sample solution, Ab_2_ = absorbance of mixture without the enzyme, Ab_3_ = absorbance of mixture without the sample, and Ab_4_ = absorbance of mixture only starch without the sample and enzyme.

#### 4.9.2. Determination of α-Glucosidase Inhibition

The α-glucosidase inhibitory activity was evaluated using the method by Kumar et al. [53]. Briefly, samples and standard stock solutions were prepared (10 mg/mL) and further diluted with 50 mM PBS (pH 6.9) to a final range of 0.5–5.0 mg/mL. The sample (50 μL) was mixed with α-glucosidase enzyme (50 μL, 0.57 unit/mL) dissolved in PBS (50 mM, pH 6.9) and then incubated in an orbital shaker (37 °C, 10 min). Then, 50 μL of substrate (15 mg of *p*-nitrophenyl α-d-glucopyranoside) was dissolved in 10 mL of PBS (50 mM, pH 6.9) and added to the reaction mixture. The reaction mixture was incubated again (37 °C, 20 min). The reaction was then stopped by adding 1 M Na_2_CO_3_ (50 μL) and the absorbance of the solution was measured at 405 nm. The α-glucosidase inhibition was calculated and expressed as %. The 50% inhibitory concentration (IC_50_) of α-glucosidase was calculated through linear regression and the percentage of inhibition calculated according to the following equation:

% inhibition = [An − (As − Abs)/An)] × 100,(2)

where An = absorbance of negative solution (no sample), As = absorbance of sample solution, and Abs = absorbance of the blank sample solution.

### 4.10. Anti-Inflammatory Activity

#### 4.10.1. Membrane Lysis Assay

##### Preparation of Erythrocyte Suspension

A suspension of erythrocytes was prepared according to the method described Shinde et al. [54], with little modifications. Briefly, human blood sample was collected from a healthy man. The blood was centrifuged (900 rcf, 5 min) and washed three times with an equal volume of normal saline NaCl (0.9% *w*/*v*). After centrifugation, the blood volume was measured and reconstituted (10% *v*/*v*) with an isotonic buffer solution (sodium phosphate buffer, 10 mM, pH 7.4).

##### Heat-Induced Hemolysis

The heat-induced hemolysis test was performed according to the method described by Okoli et al. [43] and Gunathilake et al. [55], with little modifications. Briefly, the blood cell suspension (0.05 mL) and the algal extract (0.05 mL) were mixed with PBS (2.95 mL, pH 7.4). The reaction mixture was incubated for 20 min at 54 °C. After incubation, the mixture was centrifuged at 2500 rcf for 3 min, and the absorbance of the supernatant measured at 540 nm. PBS was used as control. The level of hemolysis was calculated by using the following equation:

% inhibition = (1 − As/Ac) × 100,(3)

where Ac = absorption of the control sample, and As = absorption of the sample.

##### Protein Denaturation Assay

Protein denaturation assay was performed according to the method described by Gambhire et al. [56], with little modifications. Briefly, the reaction mixture was prepared by adding 1% bovine serum albumin (BSA, 0.2 mL), PBS (4.78 mL, pH 6.4), and the algal extract (0.02 mL). The mixture was then incubated in a water bath (15 min, 37 °C), and the reaction mixture heated for 5 min at 70 °C. After cooling, the turbidity of the solution was measured at 660 nm. PBS was used as control. The percentage of inhibition of protein denaturation was calculated by using the following formula:

% inhibition = (1 − As/Ac) × 100,

where Ac = absorption of the control sample, and As = absorption of the sample.

##### Proteinase Inhibitory Activity

The proteinase inhibitory activity of the extracts was determined according to a method described by Sakat et al. [57], with little modification. Briefly, the reaction mixture was prepared by adding trypsin (0.06 mg), Tris-HCl buffer (1 mL, 20 mM, pH 7.4), and the algal sample (1 mL, containing 0.02 mL of algal extract and 0.980 mL of methanol). The reaction mixture was incubated for 5 min at 37 °C and then casein was added (1 mL, 0.8%, *w*/*v*). The reaction mixture was incubated again for additional 20 min at 37 °C and then 70% perchloric acid (2 mL) was added to stop the reaction. The reaction mixture was centrifuged at 2500 rcf for 5 min and the absorbance of the supernatant measured at 210 nm by using the buffer as blank. PBS was used as control. The percentage of inhibition of protein denaturation was calculated by the following formula:

% inhibition = (1 − As/Ac) × 100,

where Ac = absorption of the control sample, and As = absorption of the sample.

##### Lipoxygenase Inhibition Assay

The lipoxygenase activity of the extracts was evaluated as described by Wu et al. [58], with little modifications. Briefly, the reaction mixture containing sodium borate buffer (1 mL, 0.1 M, pH 8.8) and lipoxygenase (10 μL, final concentration 8000 U/mL) was incubated with 10 mL algal extract in a 1 mL cuvette at room temperature for 5 min (30 ± 2 °C). The reaction was initiated by the addition of linoleic acid (10 μL, 10 mmol). The absorbance of the reaction mixture was then measured at 234 nm. PBS was used as control, and the percentage of inhibition of the lipoxygenase activity was calculated by using the following equation:

% inhibition = 100 × (Ac − As)/Ac,(4)

where Ac = absorbance of the control, As = absorbance of the sample.

### 4.11. Statistical Analysis

All experiments were carried out in triplicate and were expressed as mean ± standard deviation (SD). IC_50_ values were calculated by fitting the data through linear regression. The statistical significance between the obtained values (extract vs. standard) was assessed through a one way analysis of variance (ANOVA) (*, ^+^, ^&^, ^#^, ^$^ for *p*-values between 0.05 and 0.005; **, ^++^, ^&&^, ^##^, ^$$^ for *p*-values below 0.005). The correlation between TPC and TAC was estimated through linear regression and the resulting coefficient (R^2^) was used to establish the goodness of the fit. All the statistical analyses were performed by using GraphPad Prism 5 software (version 5.00, GraphPad Software Inc., La Jolla, CA).

## 5. Conclusions

Polyphenols present in the secondary metabolites from plant are gaining an even increasing attention because of their antioxidant effect. In this regard, marine algae represent a huge resource of secondary metabolites, although more studies are still needed to investigate their properties.

In this study secondary metabolites from *Enteromorpha intestinalis* were extracted by using three solvents with different polarity and physico-chemical characteristics, namely methanol, ethanol, and hexane. The extracts, especially that from the protic solvents, contained substantial amounts of phenols and flavonoids (up to about 60 and 625 mg of GAE/g and RUE/g, respectively, in the extract from ethanol), which were able to significantly reduce oxidative stress in vitro. The extract from ethanol was able to reduce hydrogen peroxide and superoxide anion radicals with IC_50_ values (about 3.2 and 2.7 μg/mL, respectively) almost comparable to those obtained from ascorbic acid (about 3.1 and 2.3 μg/mL, respectively). On the other hand, the extract from methanol yielded even better IC_50_ values in the DPPH^•^ and hydroxyl radical scavenging assays (about 0.5 and 0.3 μg/mL, respectively) compared to AA (about 2.6 and 2.3 μg/mL, respectively). This antioxidant activity of the extracts also induced a potential antidiabetic efficacy, with the methanolic extract inhibiting α-amylase and α-glucosidase (IC_50_ of about 3.8 μg/mL in both cases) almost as well as acarbose (IC_50_ of about 2.8 and 3.3 μg/mL, respectively), a commercial antidiabetic drug. Similarly, the extract from methanol presented marked anti-inflammatory effect, as demonstrated by inhibition of hemolysis, protein denaturation, proteinase activity, and lipoxygenase activity, with IC_50_ values (about 10.8, 3.7, 5.8, and 4.7 μg/mL, respectively) almost as low as those obtained with acetylsalicylic acid (about 8.6, 3.1, 5.1, and 4.5 μg/mL, respectively).

With this strong antioxidant efficacy, the extracts, and in particular that from the alcoholic solution, could potentially represent valuable coadjuvants in the treatment of diabetes and other inflammatory diseases. However, additional studies are still required for isolating and identifying the many secondary metabolites responsible for the antioxidant effects, and for addressing their role in the protective action. Although this study represents just a preliminary starting point, the promising results obtained evidence the high potentiality of the secondary metabolites contained in the *E. intestinalis*. Future studies with a more detailed characterization of the extract, e.g., by NMR and HPLC, as well as of its biological effects, e.g., in cellular and animal studies, could eventually lead to the development of dietary supplements with, e.g., anti-aging activity, and/or to the discovery of novel drugs or prototypes with pharmacological effect toward oxidative stress-related diseases.

## Figures and Tables

**Table 1:** Presence (+) or absence (−) of several phytochemical classes in *Enteromorpha intestinalis* extracts using different solvents.

Bioactive Compounds	MEEI	EEEI	HEEI
Alkaloids	+	+	+
Glycosides	+	+	+
Reducing sugars	−	−	−
Proteins	+	+	+
Terpenoids	+	+	+
Phenols and Tannins	+	+	+
Steroids	−	−	−
Saponins	+	−	−
Anthocyanins	−	−	−
Coumarin	+	+	+

**Table 2:** Functional groups and bonding patterns in the methanolic, ethanolic, and hexane extracts of *Enteromorpha intestinalis* as determined by FT-IR analysis.

Extract	Peak Value (cm^−1^)	Functional Group	Bonding Pattern
MEEI	3726.47	Alcohol (free)	O-H stretching
	2920.23	Alkane	C-H stretching
	2850.79	Alkane	C-H stretching
	2358.94	Carbon dioxide	O=C=O
	1743.65	Esters (6-membered lactone)	C=O stretching
	1031.92	Sulfoxide	S=O stretching
	669.30	Halo compound	C-Br
EEEI	3354.21	Alcohol (intermolecular bond)	O-H stretching
	2922.16	Alkane	C-H stretching
	2850.79	Alkane	C-H stretching
	2358.94	Carbon dioxide	O=C=O
	1643.35	Alkene (cis-disubstituted)	C=C stretching
	1045.42	Sulfoxide	S=O stretching
	667.37	Halo compound	C-Br
HEEI	3726.47	Alcohol (free)	O-H stretching
	2922.16	Alkane	C-H stretching
	2852.72	Alkane	C-H stretching
	2358.94	Carbon dioxide	O=C=O
	1710.86	Conjugated acid	C=O stretching
	1462.04	Sulfate	S=O stretching
	1259.52	Alkyl aryl ether	C-O stretching
	750.31	Monosubstituted	C-H bending
	669.30	Halo compound	C-Br

**Table 3:** Total phenolic, flavonoid, and ascorbic acid content, and total antioxidant capacity in *Enteromorpha intestinalis* extracts.

Assay	MEEI	EEEI	HEEI
Total phenolic content (mg of GAE ^1^/g)	23.00 ± 0.05 *	59.67 ± 0.01 **^++^	11.91 ± 0.06 *^+^
Total flavonoid content (mg of RUE ^2^/g)	416.00 ± 0.03 ^##^	624.67 ± 0.05 ^##++^	152.00 ± 0.14 ^#+^
Ascorbic acid content (µg of AA/g)	336.00 ± 0.36 ^$^	446.00 ± 0.43 ^$$++^	195.00 ± 0.33 ^$+^
Total antioxidant activity (mg of AAE ^3^/g)	88.00 ± 0.02 ^&^	177.33 ± 0.02 ^&&++^	45.00 ± 0.04 ^&+^

^1^ GAE: gallic acid equivalents; ^2^ RUE: rutin equivalents; ^3^ ascorbic acid equivalents. Relative levels of significance were determined by comparing the extracts to respective standards in the assays; * represents the significance of TPC with gallic acid, ^#^ represents the significance of TFC with rutin, ^$^ represents the significance of AA content with ascorbic acid, ^&^ represents the significance of TAA with ascorbic acid equivalent. The ^+^ represents level of significance between either EEEI or HEEI with respect to MEEI.

**Table 4:** IC_50_ values (in µg/mL) obtained from the free radical scavenging assays with ascorbic acid and the *Enteromorpha intestinalis* extracts.

Assay	Ascorbic Acid	MEEI	EEEI	HEEI
DPPH^•^ scavenging activity	2.62 ± 0.23	0.50 ± 0.01 **	3.38 ± 0.57 *	3.68 ± 0.98 *
H_2_O_2_ scavenging activity	3.13 ± 0.87	4.72 ± 0.67 *	3.22 ± 0.68 *	18.41 ± 1.78 **
Hydroxyl radical scavenging activity	2.26 ± 0.22	0.32 ± 0.01 **	2.58 ± 0.11 *	2.68 ± 0.87 *
Superoxide scavenging activity	2.26 ± 0.67	2.49 ± 0.32 *	2.73 ± 0.16 *	3.69 ± 0.73 *

Relative levels of significance were determined by comparing the extracts to ascorbic acid.

**Table 5:** IC_50_ values (in µg/mL) of the antidiabetic activity of the extracts of *Enteromorpha intestinalis*.

Assay	Acarbose	MEEI	EEEI	HEEI
α-Amylase activity	2.81 ± 0.78	3.81 ± 0.45 *	4.15 ± 0.56 *	4.68 ± 0.33 *
α-Glucosidase activity	3.32 ± 0.96	3.82 ± 0.56 *	4.32 ± 0.78 *	5.44 ± 0.51 *

Relative levels of significance were determined by comparing the extracts to acarbose.

**Table 6:** IC_50_ values (in µg/mL) of the anti-inflammatory activity of the extracts of *Enteromorpha intestinalis*.

Assay	Acetylsalicylic Acid	MEEI	EEEI	HEEI
Hemolysis inhibition activity	8.62 ± 0.99	10.81 ± 1.12 *	11.21 ± 1.76 *	13.09 ±1.98 *
Protein denaturation activity	3.14 ± 0.88	3.68 ± 0.81 *	3.95 ± 0.19 *	4.88 ± 0.49 *
Proteinase activity	5.11 ± 0.97	5.84 ± 0.91 *	6.89 ± 0.37 *	7.60 ± 0.12 *
Lipoxygenase activity	4.52 ± 0.58	4.72 ± 0.76 *	4.95 ± 0.86 *	6.01 ± 0.27 *

Relative levels of significance were determined by comparing the extracts to ASA.

## Data Availability

Raw data are available upon request from the authors.
